# Identification, Characterization, and Cytological Analysis of Several Unexpected Hybrids Derived from Reciprocal Crosses between Raphanobrassica and Its Diploid Parents

**DOI:** 10.3390/plants12091875

**Published:** 2023-05-04

**Authors:** Jie Yu, Shaolin Lei, Shiting Fang, Niufang Tai, Wei Yu, Ziwei Yang, Lei Gu, Hongcheng Wang, Xuye Du, Bin Zhu, Mengxian Cai

**Affiliations:** 1 School of Life Sciences, Guizhou Normal University, Guiyang 550025, China; 21010100402@gznu.edu.cn (J.Y.); 190902010054@gznu.edu.cn (S.F.); 201109040009@gznu.edu.cn (N.T.); 201109040010@gznu.edu.cn (W.Y.); 211109030172@gznu.edu.cn (Z.Y.); 201808009@gznu.edu.cn (L.G.); wanghc@gznu.edu.cn (H.W.); duxuye@gznu.edu.cn (X.D.); 2 Guizhou Institute of Oil Crops, Guizhou Academy of Agricultural Sciences, Guiyang 550009, China; leisl66@126.com

**Keywords:** *Brassica oleracea*, radish, interspecific hybridization, unexpected hybrids, chromosome behaviour

## Abstract

Interspecific hybridization and accompanying backcross between crops and relatives have been recognized as a powerful method to broaden genetic diversity and transfer desirable adaptive traits. Crosses between radish (*Raphanus sativus*, RR, 2n = 18) and *Brassica oleracea* (CC, 2n = 18), which formed allotetraploid Raphanobrassica (RRCC, 2n = 36), initiated the construction of resynthetic allopolyploids. However, these progenies from the backcrosses between Raphanobrassica and the two diploid parents have not been well deciphered. Herein, thousands of backcrosses using both Raphanobrassica and the two diploid parents as pollen donors were employed. Several hybrids with expected (2n = 27) and unexpected chromosome numbers (2n = 26 and 2n = 36) were obtained. Fluorescence in situ hybridization (FISH) analysis with R-genome-specific sequences as probes demonstrated that the genome structures of the two expected hybrids were RRC and CCR, and the genome structures of the three unexpected hybrids were RRRC, CCCR, and RRC’ (harbouring an incomplete C genome). The unexpected hybrids with extra R or C genomes showed similar phenotypic characteristics to their expected hybrids. FISH analysis with C-genome-specific sequences as probes demonstrated that the unexpected allotetraploid hybrids exhibited significantly more intergenomic chromosome pairings than the expected hybrids. The expected and unexpected hybrids provide not only novel germplasm resources for the breeding of radish and *B. oleracea* but also very important genetic material for genome dosage analysis.

## 1. Introduction

Interspecific hybridization, which contributes to the current allopolyploid formation, has been considered a driving force for abrupt speciation and has occurred extensively in flowering plants [1,2,3]. Recently, because crops have been challenged by a severe continuous decline in genetic diversity due to domestication that has continued over several decades of an extensive selection of modern breeding, interspecific hybridization between cultivars, and their relatives has been widely used to introgress desirable adaptive traits [4,5,6,7,8,9]. However, hybrids from interspecific hybridization are generally infertile [10]. Even though they generate progeny after selfing, these progenies are unable to be directly applied in crop breeding due to extensive and disordered variations [11]. Interspecific or intergeneric backcrossing using receptors as pollen donors has been recognized as an effective strategy to transfer desirable traits [5,9,10,12,13].

Homoeologous chromosome recombination between donor and receptor chromosomes, which presents directly through the formation of crossovers between homoeologous chromosomes at meiosis and occurs at low frequency, is another prerequisite for successfully recovering the desirable trait in segregating progenies [14]. Assessing homoeologous chromosome recombination in hybrids can provide knowledge about approximately how many subsequent generation plants will be prepared to obtain the desired introgression [15]. It is believed that homoeologous recombination is restricted by at least two mechanisms: (1) the physical distinction between these homoeologous pairs and (2) some genetic control factors of chromosome pairing [16]. In allohexaploid wheat (AABBDD, 2n = 42), the *ph1* system, which prevents nonhomologous recombination, has been well deciphered [17,18]. To overcome the suppression of homoeologous recombination, wheat lines with inactivation or deletion of *ph1* were involved in hybridizations between wheat and its relatives. A similar genetic locus (*PrBn*) controlling homoeologous recombination was detected in *Brassica napus* (AACC, 2n = 38) [19]; however, this locus remains undescribed. Additionally, Cui et al. [20] reported that genome combinations and the cytoplasmic background significantly affected homoeologous chromosome pairing after analysing a series of *Brassica* hybrids.

Brassicaceae, which comprises 338 genera and 3709 species, is a quite large family in angiosperm [21]. *Brassica* genus is the most important genus in this family because it comprises numerous commercial crops [22]. These crops can be represented by six interrelated species, or the “U” triangle [23]. The U triangle comprises three diploid ancestors, *Brassica rapa* (AA, 2n = 20), *B. oleracea* (CC, 2n = 18), and *B. nigra* (BB, 2n = 16), and three allotetraploid species, *B. napus*, *B. juncea* (AABB, 2n = 36), and *B. carinata* (BBCC, 2n = 34), derived from the interspecific crosses of any two of the three diploid species.

*Raphanus sativus* (RR, 2n = 18) (radish) is another economically important species in Brassicaceae owing to its edible roots. *Brassica* species and *R. sativus* share a very close phylogenetic relationship [24,25]. Additionally, radish is tolerant to several diseases that usually cause severe yield loss in *Brassica* crops [26,27,28]. Therefore, interspecific crosses involving *Brassica* species and radish that aim to create novel allopolyploids or to transfer desirable traits have been performed for approximately 200 years [7,8,28,29,30,31,32]. Sageret attempted to synthesize intergeneric hybrids between radish and *Brassica* species in 1826 [31] which initiated the construction of intergeneric hybrids in Brassicaceae. Crucifer club root, a soil-borne disease, which is caused by the plant pathogen *Plasmodiophora brassicae*, has been a serious threat to the rape industry in the world [33]. Interspecific crosses between resynthetic Raphanobrassica (RRCC, 2n = 36) and *B. napus* and the subsequently continuous backcrosses could successfully transfer the club root tolerance into *B. napus* [28]. As the most important achievement in interspecific hybridization in *Brassica*, the Ogura cytoplasmic male sterility (CMS) system was successfully obtained to introduce sterility and restorer genes into *B. napus* using an interspecific cross between *B. napus* and radish [34,35]. This CMS system has been successfully and extensively used to produce hybrid rape seed. Furthermore, Brassicoraphanus hybrid (AARR, 2n = 38) from the interspecific cross between *B. rapa* and radish has been recently generated for genetic study and improvement [32,36].

In our previous study, to enhance the *Brassica* species’ genetic diversity, a Raphanobrassica was reconstructed through the interspecific hybridization between maternal radish and paternal *B. oleracea* [8]. Herein, backcrosses using both Raphanobrassica and the two diploid parents as pollen donors were employed. Intriguingly, not only two expected hybrids (genome structure: RRC and CCR) but also three unexpected hybrids (genome structure: RRRC, CCCR, and RRC’, harbouring an incomplete C genome) that were reported for the first time, to the best of our knowledge, were obtained after checking by fluorescence in situ hybridization (FISH) analysis. The characteristics, genome constructure, and chromosome pairing of these hybrids were well addressed in this study. We believe that these hybrids can be used as novel germplasm resources for breeding and genetic analysis of radish and *B. oleracea*.

## 2. Results

### 2.1. Development of Hybrids from Reciprocal Crosses

**Figure 1:**
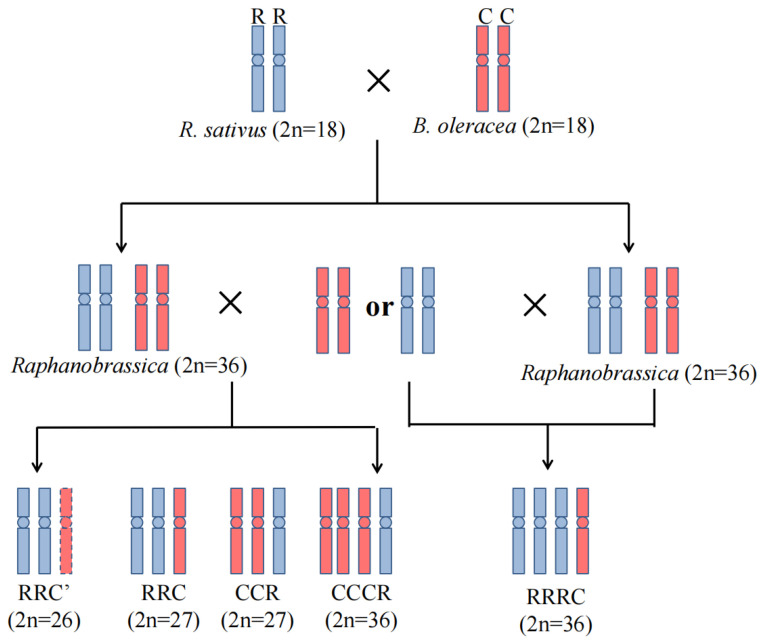
Elementary diagram of the production of the triploid and unexpected hybrids. The diagrams with blue and red colours represent R and C genomes, respectively.

After pollinating hundreds of flowers for crosses (432 and 548 emasculated flower buds for the combinations of RRCC × CC and RRCC × RR) and reciprocal crosses (523 and 174 emasculated flower buds for the combinations of CC × RRCC and RR × RRCC) between allotetraploid Raphanobrassica and its diploid parents, 14 embryos were obtained with ovary culture and subsequent embryo rescue (Figure 1, Table 1). Among these embryos, one (0.57% of pollinated flowers) embryo was obtained from the cross of RR × RRCC, three (0.69%) embryos from the cross of RRCC × CC, and ten (1.82%) embryos from the cross of RRCC × RR, whereas no embryos were generated from the cross combination of CC × RRCC. More than 10 cloned plantlets per embryo were cultured using MS medium. After counting the chromosome number of these hybrids, only four hybrids had an expected chromosome number (2n = 27) (two hybrids from RRCC × CC and two hybrids from RRCC × RR). Intriguingly, two hybrids (one from RR × RRCC and one from RRCC × CC) had a chromosome number of 2n = 36 but showed obviously different phenotypes in comparison with the parent Raphanobrassica (RRCC), indicating that the two hybrids were unexpected hybrids. Additionally, one hybrid from RRCC × RR had a chromosome set of 2n = 26, likely owing to unstable chromosome pairing at meiosis of Raphanobrassica and exhibited characteristics similar to those of the expected hybrids.

### 2.2. Determination of Chromosome Constitutions of the Unexpected Hybrids

**Figure 2:**
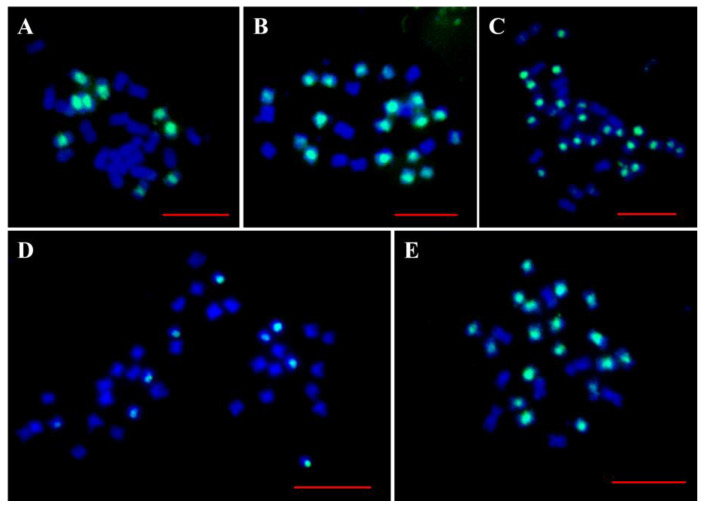
FISH analyses of chromosome constitutions of the triploid and unexpected hybrids. The blue signal indicates DAPI staining, and green signal represents the specific satellite repeat sequences of R genome. (**A**) Metaphase cell of triploid CCR harbouring 18 C chromosomes (blue) and 9 R chromosomes (green). (**B**) Metaphase cell of triploid RRC with 18 R chromosomes (green) and 9 C chromosomes (blue). (**C**) Metaphase cell of unexpected hybrid RRRC with 9 C genome chromosomes (blue) and 27 R genome chromosomes (green). (**D**) Metaphase cell of unexpected hybrid CCCR with 27 C chromosomes (blue) and 9 R genome chromosomes (green). (**E**) Metaphase cell of unexpected hybrid RRC’ with 8 C chromosomes (blue) and 18 R chromosomes (green). Bar, 10 μ.

To determine the chromosome constitution of these expected and unexpected hybrids, FISH using the R genome-specific sequence (repeat sequences CL1) of *R. sativus* [37] as a probe was applied to these hybrids. As expected, the results showed that the expected hybrids from RRCC × CC had nine green signals (representing the R chromosome, Figure 2A), and the expected hybrids from RRCC × RR had 18 green signals (Figure 2B), indicating that the genome constitutions of the two types of hybrids were CCR and RRC. For the unexpected hybrid with 2n = 36 from RR × RRCC, 27 green signals were detected (Figure 2C), indicating that this hybrid harboured three R genomes and one C genome (RRRC). For another unexpected hybrid with 2n = 36 from RRCC × CC, nine green signals were observed (Figure 2D), suggesting that the genome constitution of this hybrid was CCCR. Eighteen green signals and eight blue C chromosomes stained with DAPI (Figure 2E) were detected in the hybrid with 2n = 26 from RRCC × RR, suggesting that one of the C chromosomes was missing from this hybrid (RRC’). The FISH results of these hybrids were listed in (Table 2).

**Figure 3:**
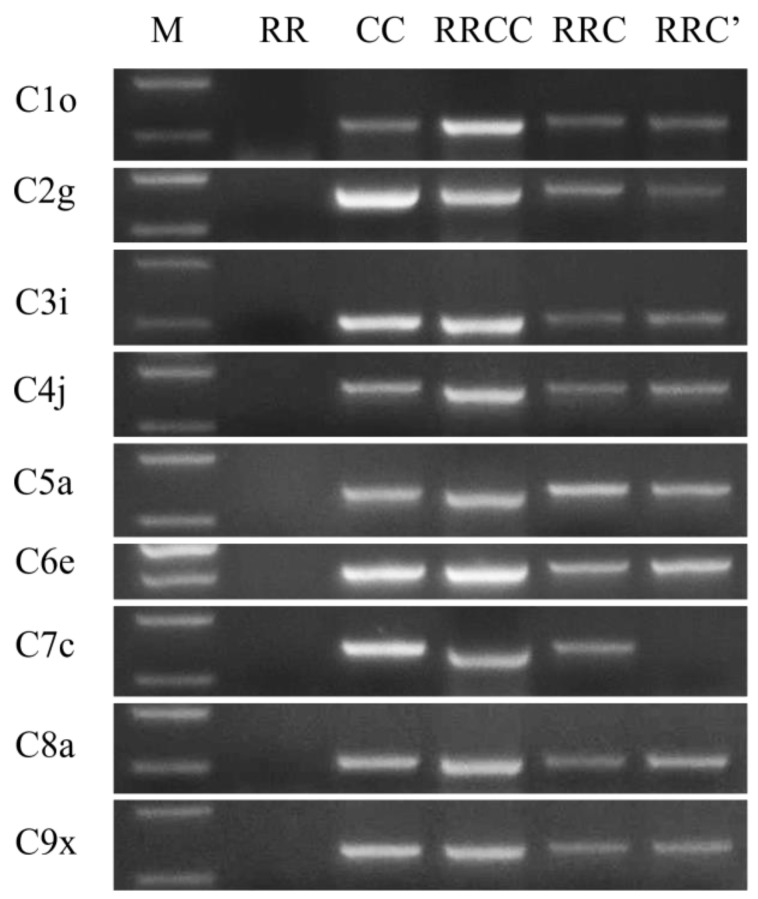
Identification of unexpected hybrid RRC’ using PCR screening of C genome-specific primers. M represents markers used in this study (DM2000, CWBIO).

To confirm which C chromosome was missing from the RRC’ hybrid, a complete set of C chromosome-specific primers [4] was used to screen this hybrid. The results showed that the amplified products of all chromosome-specific primers except the primer for chromosome C7 were amplified in this hybrid (Figure 3), indicating that chromosome C7 was missing from this hybrid.

### 2.3. Morphology and Fertility of the Unexpected Hybrids

**Figure 4:**
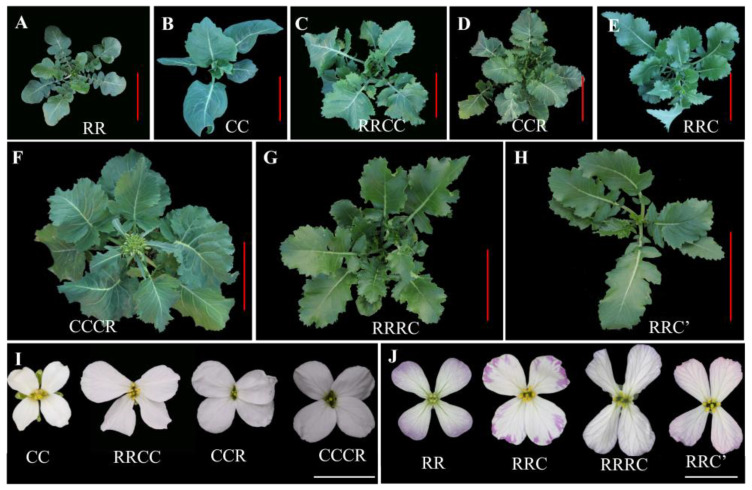
Morphological analysis of parental *R. sativus*, *B. oleracea*, synthetic Raphanobrassica, and their hybrids. (**A**–**H**) The phenotype of seedlings of *R. sativus*, *B. oleracea*, synthetic Raphanobrassica, triploid CCR, triploid RRC, CCCR hybrid, RRRC hybrid, and RRC’ hybrid. Bar, 15 cm. (**I**) The morphology of flowers of *B. oleracea*, synthetic Raphanobrassica, triploid CCR, and unexpected hybrid CCCR. Bar, 1 cm. (**J**) The morphology of flowers of *R. sativus*, triploid RRC unexpected hybrid RRRC, and RRC. Bar, 1 cm.

The seedling phenotypes of these hybrids and parents were well documented (Figure 4A–H). Generally, the hybrids exhibited a seedling phenotype and flower type (Figure 4I,J) biased toward that of the parent with the dominant genome. Briefly, compared to parental radish (Figure 4A) and *B. oleracea* (Figure 4B), the allotetraploid RRCC (Figure 4C) showed a phenotype biased toward that of *B. oleracea* with ceraceous, thick leaves, and white flowers. Similar to allotetraploid RRCC, the CCR hybrid, which had the earliest flowering time among these hybrids, exhibited thick and waxy leaves and large and white flowers but a smaller plant architecture than RRCC (Figure 4D). The RRC plant showed plant architecture and morphology biased towards these paternal radishes with ultra hairy and marginally dentate leaves and especially spotted purple flowers (Figure 4E,J) but no inflated roots. Compared to its parents and other hybrids, the RRC hybrid exhibited the most delayed flowering time which was approximately 1–3 weeks later than that of other plants. The CCCR hybrids had a plant morphology similar to that of CCR; however, they exhibited larger flowers, leaves, and plant architecture (Figure 4F,I) which was attributed to genome dosage or ploidy of the C genome. The unexpected RRRC hybrid had phenotypic characteristics similar to that of RRC but exhibited larger flowers and leaves (Figure 4G) which were likely to be attributed to genome dosage or ploidy of the R genome. The RRC’ hybrid (Figure 4H) showed the weakest plant architecture, with light green leaves, probably due to the impact of genome imbalance of the incomplete C genome. All the hybrids were sub-cultured using MS medium with consideration of the wide divergence of their progenies after selfing.

### 2.4. Chromosome Behaviours of the Triploid and Unexpected Hybrids

**Figure 5:**
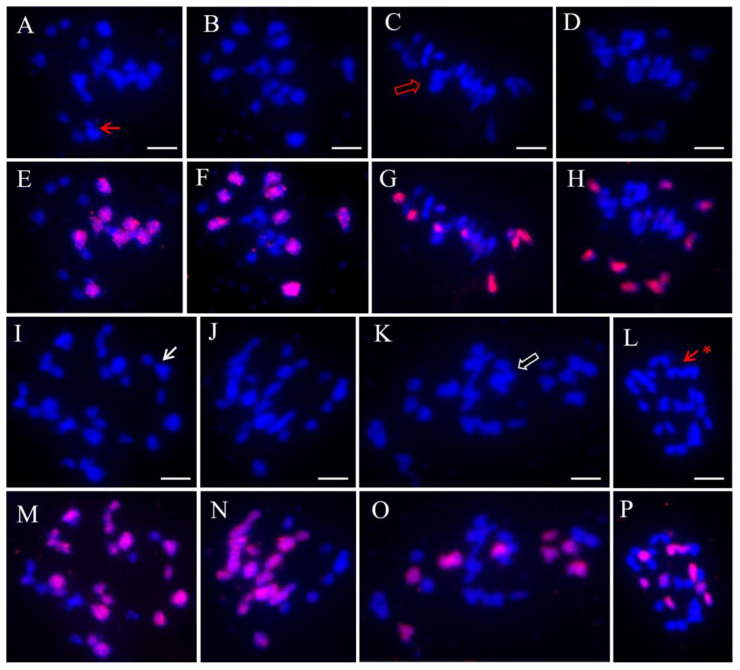
BAC-FISH analyses of chromosome behaviours of the triploid and unexpected hybrids. The blue signal indicates DAPI staining, and red indicates labelled BAC BoB014O06 specific to the *Brassica* C-genome. (**A**,**B**,**E**,**F**) PMC chromosomal configurations of triploid CCR at diakinesis. The red arrows indicate Ⅲ^C-C-R^. (**C**,**D**,**G**,**H**) PMC chromosomal configurations of triploid RRC at diakinesis. The hollow red arrow indicates IV ^R-R-C-R^. (**I**,**J**,**M**,**N**) PMC chromosomal configurations of unexpected hybrid CCCR at diakinesis. The white arrows indicate Ⅲ^C-C-R^. (**K**,**L**,**O**,**P**) PMC chromosomal configurations of unexpected hybrid RRRC at diakinesis. The hollow white arrow indicates IV^R-C-R-R^, and the red arrow with an asterisk indicates IV^C-C-R-R^. Bar, 5 μm.

The chromosome behaviours at meiosis, particularly the frequency of homoeologous chromosome pairing in diakinesis (DI), which ensures successful introgression of donor features (genes), has been the focus of most attention. In total, 17–49 unambiguous pollen mother cells (PMCs) at DI of these hybrids except RRC’ were observed by using the labelled C genome-specific (BoB014O06) probe (Figure 5). Homoeologous chromosome pairing, including allosyndesis (homoeologous chromosome pairing between different genomes) and autosyndesis types (homoeologous chromosome pairing in same genome), was proven to prevail in these PMCs (Table 3). Out of 31 PMCs of the CCR hybrid, 11 PMCs (35.48%) showed homoeologous chromosome pairing, whereas these tested PMCs of the RRC hybrid (35 of 49 PMCs, 71.43%) exhibited significantly higher homoeologous pairing than that of CCR (χ^2^-test, *p* < 0.05). Intriguingly, both unexpected hybrids showed obviously prevailing homoeologous pairing in their PMCs (15 of 17 PMCs in RRRC and 34 of 49 PMCs in CCCR). For these allosyndesis types, the homoeologous trivalent (Ⅲ) types were dominant, including Ⅲ^R-C-R^, Ⅲ^C-C-R^, Ⅲ^C-R-C^, and Ⅲ^R-R-C^, followed by the homoeologous divalent (Ⅱ^R-C^). A few homoeologous quadrivalents (IV) and pentavalent (V) hybrids, which were frequent among the unexpected hybrids, were also observed. Some autosyndesis types, including Ⅱ^C-C^, Ⅱ^R-R^, Ⅲ^C-C-C^, and Ⅲ^R-R-R^, were detected.

## 3. Discussion

The allotetraploid Raphanobrassica from the intergeneric cross between a radish and *B. oleracea* has been perceived as a perfect model with which to analyse allopolyploidization in plants at different levels [38,39,40]. Moreover, Raphanobrassica has been taken as an important intermediate material to impart tolerance traits and cytoplasmic male sterility to *Brassica* species [28,30,34] through successive backcrosses. However, these backcrosses were generally performed using *Brassica* species as pollen donors.

In this study, two expected hybrids (RRC and CCR) and three unexpected hybrids, including two autoallopolyploid hybrids (RRRC and CCCR) and an aneuploid triploid (RRC’), were obtained through reciprocal backcrosses using both Raphanobrassica and the two diploid parents as pollen donors. To our knowledge, these unexpected hybrids were obtained for the first time by means of intergeneric crosses between allotetraploid Raphanobrassica and diploid parents. A similar result was observed in the work of Cui et al. [20], in which three unexpected triploids (genome constitution: BBA, CCA, and CCB) from reciprocal pair crosses of three *Brassica* diploids were obtained. Cui et al. [20] speculated that these unexpected hybrids probably resulted from the fusion of a female unreduced gamete (gametes with the somatic chromosome number) and a normal male gamete, alternatively attributed to genome duplication for one genome doubled during the mitotic divisions of the zygote after fertilization. One unexpected hybrid with the genome constitution of ACRR from the interspecific hybridization between transgenic *B. napus* and wild radish was also generated which was believed to be attributed to the unreduced gamete of radish [41]. The frequency and maintenance of unreduced gametes in allopolyploid *Brassica* species have been systematically analysed, but the frequency of unreduced pollen was only estimated to range from 0.0% to 0.11% [42]. It has been reported that diploid *B. oleracea* and radish can also generate unreduced gametes [41,43]. However, the occurrence of unreduced gametes in these two species has not been estimated. After checking hundreds of pollen grains from diploid parents, we did not detect any giant pollen grains that are generally recognized as unreduced male gametes in the two diploid parents (data not shown), indicating that the occurrence of unreduced gametes is rare. In the present study, the combination of RR × RRCC generated a unique RRRC hybrid (0.57% of pollinated flowers), and the combination of RRCC × CC gave rise to two expected hybrids and one unexpected hybrid (0.23% of pollinated flowers). Obviously, the frequency of unexpected hybrids was higher than the occurrence of unreduced gametes, indicating that these unexpected hybrids are unlikely attributed to the unreduced gametes, only when these unreduced gametes were preferentially selected during interspecific hybridization. Therefore, we supposed that the single genome duplication during the mitotic divisions of the zygote after fertilization was likely involved in the formation of these unexpected hybrids.

Meiotic homoeologous chromosome pairing is an important aspect of interspecific hybridization because it is a prerequisite for ensuring introgression between the receptor and donor genomes [15]. Knowledge of the occurrence frequency of homoeologous chromosome pairing of interspecific hybrids is invaluable in not only predicting the feasibility of introgression of desirable traits before successive backcrosses but also knowing approximately how large a population needs to be in order to guarantee the success of the desired introgression [15]. In natural alloploids, homoeologous chromosome pairing is restricted by some genetic control factors, such as *ph1* and *ph2,* in wheat [17,18] and *PrBn* loci in *B. napus* [16,19,44]. The homoeologous chromosome pairing in synthetic interspecific hybrids may be affected by the physical divergence between homoeologous chromosomes [16,20], the genome combinations, and the cytoplasmic background that these hybrids inherited [20]. Distinct homoeologous chromosome pairings are also observed for the present hybrids which have the same cytoplasmic background (with the cytoplasm of all the hybrids inherited from radish), and the genomes with the same constitution differ in copy number. The hybrids that harbour the dominant R genome (RRC and RRRC) likely show more prevailing homoeologous pairings than the hybrids harbouring the dominant C genome (CCR and CCCR) (Table 3), indicating that genome dosage is probably involved in chromosome pairing.

## 4. Materials and Methods

### 4.1. Plant Materials and Crosses

Three plant cultivars or accessions were used in the present study, including the diploid *B. oleracea* cultivar ‘Chijielan’ and *R. sativus* cultivar ‘Loutouqing’ and a recently resynthesized allotetraploid Raphanobrassica derived from an interspecific cross between the above two diploid parents [8]. These materials were initially germinated in small pots (3 × 3 cm in width and length and 5 cm in depth) in a growth cabinet under a condition of 16:8 light–dark cycles with a constant temperature of 24 °C and a relative humidity of 40%. When the seedlings had three true leaves, they were transferred into larger pots (50 in diameter and 40 cm in depth) in the experimental field under the same cultivation conditions at Guizhou Normal University (Guiyang, China).

Reciprocal crosses between allotetraploid Raphanobrassica and its two diploid parents were employed (Figure 1). To reduce cross-incompatibility, the maternal plants were artificially pollinated after their flowers were castrated for more than 24 h. In total, more than one thousand flowers were castrated. The siliques that showed obvious expansion were cultured in an MS medium for 10 days after pollination. Then, embryo rescue was used to obtain hybrid seedlings when these siliques were cultured in MS medium for three weeks. For further analysis, the hybrid seedlings were transferred to MS medium containing 1.0 mg/L 6-BA (6-benzyladenine) and 0.25 mg/L NAA (naphthylacetic acid) to produce cloned plants. These cloned plantlets were cultivated under the same conditions as their parents.

### 4.2. PCR Screening of C Chromosome-Specific Primers

To identify true hybrid seedlings and assess the integrity of true hybrids, PCR amplifications using the complete set of C-genome-specific primers [4] were performed. The CTAB method was employed to isolate total DNA from the young leaves. The PCR mixture in a total volume of 10 µL contained 2 µL of 50 ng genomic DNA, 4 µL of 1× Taq buffer, 1 µL of 5 mM forwards and reverse primers, 1 µL of 2.5 mM deoxy-ribonucleoside triphosphates, 1 µL of 2 mM MgCl2, and 1 µL of 0.35 U Taq DNA polymerase. PCR products were initially denatured at 94 °C for 5 min, then amplified using the following system: 30 cycles of 94 °C for 30 s, 55–57 °C for 30 s, and 72 °C for 45 s, and a final 10 min elongation step at 72 °C. Then, these PCR products were screened by 1% agarose gel electrophoresis.

### 4.3. Morphological and Cytological Analysis of These Hybrids

To check the chromosome number of these hybrids, numerous ovaries from young flower buds were collected and immediately dealt with 2 mM 8-hydroxyquinoline for 2–3 h at 22 °C in an incubator in the dark. Then, these treated ovaries were transferred to Carnoy’s liquid (mixture of ethanol and glacial acetic acid with *v/v* of 3:1) and reposited at −20 °C for further use. For chromosome behaviour detection, abundant unopened flower buds from these hybrid plants were gathered and dealt with Carnoy’s liquid until these flower buds were absolutely discoloured. The analyses of chromosome number and chromosome behaviour were performed according to the method described by Li et al. [45]. The pollen viability of these hybrids was based on the result of pollen stainability, which was determined by staining more than 300 pollen grains per plant with 1% acetocarmine. Cytological images were captured employing a computer-assisted microscope harboring a CCD camera (E200, Nikon, Japan).

### 4.4. Probe Preparation and Fluorescence In Situ Hybridization (FISH) Analysis

To decipher the genome structure and detect the chromosome behaviour of these expected and unexpected hybrids, FISH analyses with C and R genome-specific probes were used. The plasmid DNA harboring the *Brassica* C-genome specific sequence of BAC BoB014O06 [46], which gives rise to a GISH-like pattern (provided by professor Zaiyun Li, Huazhong Agriculture University, Wuhan, China), was labelled with biotin-11-dCTP BioPrime DNA Labelling System kit (Invitrogen, Life Technologies, Waltham, MA, USA) by random priming method base on the manufacturer’s protocol. To discriminate R chromosomes, plasmid DNA of BAC CL1 with an insertion sequences of 177-bp satellite repeat of *R. sativus* [37] was constructed by Sangon Biotech (Shanghai, China) and then labelled with digoxigenin-11-dUTP (Roche, Basel, Switzerland) using the BioPrime Array CGH Genomic Labelling System kit (Invitrogen, Life Technologies) through random priming method according to the manufacturer’s protocol. The chromosome slides for FISH were conducted based on the methods described by Zhong et al. [47], and the FISH methods followed the work of Cui et al. [20] with minor modifications. Briefly, we reduced the washing time to 6 min in 0.1× SSC with 20% deionized formamide. FISH images were taken using a fluorescence microscope with a CCD camera (N80i, Nikon, Japan). The merged pictures were analysed using Adobe Photoshop software (version 7.0), and only the contrast and brightness were adjusted for these pictures.

## 5. Conclusions

In this study, reciprocal backcrosses between Raphanobrassica and its two parents were employed to construct backcross hybrids. Intriguingly, two expected hybrids (RRC and CCR) and three unexpected hybrids (RRRC, CCCR, and RRC’), which are reported for the first time to the best of our knowledge, were obtained by using FISH analysis with R-genome-specific sequences as probes. Subsequently, FISH analysis with C-genome-specific sequences as probes demonstrated that the unexpected hybrids exhibited significantly more intergenomic chromosome pairings than the expected hybrids, suggesting that these unexpected hybrids can be used as potential intermediate resources for the genetic improvement of radish and *B. oleracea*. We think these hybrids can provide novel germplasm resources for breeding and genetic analysis of radish and *B. oleracea*.

## Figures and Tables

**Table 1:** Summary of interspecific hybrid embryos from different cross combinations in this study.

Cross Combinations	Emasculated Flowers Number	Obtained Embryos (%)	Genome Constitution of Embryos (%)
CC × RRCC	523	0	—
RR × RRCC	174	1 (0.57)	RRRC (0.57)
RRCC × CC	432	3 (0.69)	2 CCR (0.46); 1 CCCR (0.23)
RRCC × RR	548	10 (1.82)	2 RRC (0.36); 1 RRC’ (0.18); 7 RRCC (1.28)
Total	1677	14 (0.83)	—

RRC’ repNote: RRC’ represents that the hybrid harbors an uncomplete C genome.

**Table 2:** The chromosome constitutions of these hybrids detected by FISH.

Hybrids Types	R Chromosome Signals	C Chromosome Signals
RRC’	18	8
RRC	18	9
RRRC	27	9
CCR	9	18
CCCR	9	27

**Table 3:** Summary of homoeologous chromosome pairings in PMCs of these hybrids.

Hybrids Types	Total PMCs	Homoeologous Pairing in PMCs	Ratio	Allosyndesis Types	Autosyndesis Types
RRC^b^	49	35	71.43%	R-C; R-R-C; C-C-R; R-C-R; C-R-C; R-C-C-R; C-C-C-R; R-R-R-C;	C-C
RRRC^a^	17	15	88.23%	R-C; R-R-C; C-C-R; R-R-C-R; C-C-C-R; C-C-R-R; C-C-C-C-R;	C-C
CCR^c^	31	11	35.48%	R-C; C-C-R; C-C-R-R; R-C-C-R; C-C-C-C-R;	C-C-C; R-R-R; R-R
CCCR^b^	49	34	69.39%	R-C; R-R-C; C-C-R; R-C-R; C-R-C; C-C-C-R; C-C-R-C; C-C-C-R-R;	R-R-R

## Data Availability

Not applicable.
